# Changes in peripheral mitochondrial DNA copy number in metformin-treated women with polycystic ovary syndrome: a longitudinal study

**DOI:** 10.1186/s12958-020-00629-5

**Published:** 2020-07-13

**Authors:** Po-Kai Yang, Chia-Hong Chou, Chin-Hao Chang, Shee-Uan Chen, Hong-Nerng Ho, Mei-Jou Chen

**Affiliations:** 1 Department of Obstetrics and Gynecology, National Taiwan University Hospital, No. 8, Chung-Shan South Road, 100 Taipei, Taiwan; 2 College of Medicine, National Taiwan University, Taipei, Taiwan; 3 Department of Medical Research, National Taiwan University Hospital, Taipei, Taiwan; 4 College of Medicine, Taipei Medical University, Taipei, Taiwan; 5 Livia Shangyu Wan Scholar, National Taiwan University, Taipei, Taiwan

**Keywords:** Humans, Female, Polycystic ovary syndrome, Testosterone, Metformin, DNA mitochondrial, 8-Hydroxy-2′-Deoxyguanosine

## Abstract

**Background:**

Patients with polycystic ovarian syndrome (PCOS) are associated with known alterations in mitochondria DNA copy number (mtDNA-CN). The aim of this study is to study the change in mtDNA-CN in patients with PCOS who were treated with metformin.

**Methods:**

This is a prospective cohort of patients with PCOS, who received metformin for one year. From 2009 to 2015, 88 women diagnosed with PCOS, based on the Rotterdam criteria, were enrolled. Serial measurements of mtDNA-CN, 8-hydroxydeoxyguanosine (8-OHdG), anthropometric, metabolic, endocrine, and inflammatory markers were obtained before and after 3, 6, and 12 months of treatment.

**Results:**

A significant decrease in mtDNA-CN was seen over the course of one year. Other markers, including 8-OHdG, testosterone, free androgen index, blood pressure and liver enzymes, also decreased in the same interval. On regression analysis, there was a significant association between the change in mtDNA-CN and serum total testosterone, and no association between mtDNA-CN and metabolic factors.

**Conclusions:**

Treatment with metformin is associated with a time-dependent decrease in mtDNA-CN in patients with PCOS who are treated over the course of one year. This may signify a reduction in mitochondria dysfunction. The change in mtDNA-CN corresponds to a similar change in serum total testosterone, and suggests a possible relationship between mtDNA-CN and testosterone.

**Trial registration:**

ClinicalTrials.gov, NCT00172523. Registered September 15, 2005

## Background

Polycystic ovary syndrome (PCOS) is a common endocrine disorder in women of reproductive age and is characterized by chronic anovulation, infertility, hyperandrogenism, insulin resistance and metabolic syndrome [1]. In addition to metabolic derangements, there is increased production of reactive oxygen species (ROS) [2]. As oxidative damages from ROS are related to cardiovascular diseases and metabolic syndromes [3], this may explain the high prevalence of hyperlipidemia, hypertension, and steatohepatitis in patients with PCOS [4, 5].

Mitochondria functions, which are related to the production of ROS [6], have been assessed in patients with PCOS. Studies on leukocyte-isolated mitochondrion from patients with PCOS have shown reduced oxidative capacity [7], and decreased mitochondria mass [8]. Mitochondria DNA copy number (mtDNA-CN) are also reduced in these patients [9]. Alterations in mtDNA-CN, which may be due to increased mitochondria replication [10, 11] or destruction [12], represent functional disturbances of the mitochondria, and have been linked to a number of diseases [13, 14]. The existance of alterations in mtDNA-CN in patients with PCOS [9] suggests an underlying dysfunction in the mitochondrion of these patients.

Metformin, an insulin sensitizer primarily used in the treatment of type 2 diabetes, has been employed as treatment for patients with PCOS. It has been linked to the correction of several clinical abnormalities, including metabolic, hormonal, ovulatory, and menstrual dysfunctions [15–17]. It also reduces the ROS production from inhibition of complex I in the mitochondria [18–20]. In women with PCOS, metformin has resulted in reduced oxidative markers [21–23], improved mitochondrial respiration capacity, energy utilization, and mitochondrial mass [8]. However, there has been no study on the change in mtDNA-CN in patients with PCOS that were treated with metformin.

This study investigates the change in mtDNA-CN in metformin-treated patients with PCOS over 1 year. Changes in 8-hydroxy-2-deoxyguanosine (8-OHdG), a marker of oxidative DNA damage [24], and metabolic, anthropometric, and hormonal markers were evaluated. The change in mtDNA-CN was evaluated in association with the changes in other clinical markers, to assess for possible interactions. To the best of our knowledge, this is the first human study to evaluate the change in mtDNA-CN in metformin-treated patients.

## Methods

### Study subjects

Eighty-eight women with PCOS were enrolled into the study at the National Taiwan University Hospital from 2009 to 2015. All patients were enrolled from the reproductive endocrinology clinic with complaints of menstrual irregularities or hyperandrogenism. Diagnosis of PCOS was based on the Rotterdam criteria [25]. Other endocrine dysfunction such as hyperprolactinemia, thyroid dysfunction, Cushing syndrome, congenital adrenal hyperplasia, adrenal tumors, and virilizing tumors were excluded. Patients medicated with hormones, chemotherapy or immunosuppressive agents in the past 6 months were excluded. All major systemic diseases, including autoimmune diseases, central nervous system diseases, and malignancy were excluded. The Institutional Review Board of the National Taiwan University Hospital approved this study. Signed informed consents were obtained from all patients or their legal guardians before data collection.

### Protocol and data collection

Patients received evaluation for hormonal, metabolic, anthropometric, hepatic, inflammatory, oxidative markers and blood pressures at baseline (M0). Body mass index (BMI) was calculated as weight (kg) divided by squared height (m^2^). Overweight was defined as a BMI greater than 23 kg/m^2^, based on the WHO recommendations for Asians [26], and abdominally obese was defined as a waist circumference ≥ 80 cm [27]. Systolic (SBP) and diastolic (DBP) blood pressure were calculated as the mean of two measurements. The descriptions for blood collections have been detailed previously [28]. Briefly, the blood samples were collected after overnight fasts, and in the early follicular phase in women with spontaneous menses and immediately before hormone-induced withdrawal bleeding in amenorrheic women. A standard 75 g oral glucose test was performed at baseline, and patients were classified as normal, glucose impaired, or diabetic, based on the 2-h blood glucose cutoffs of 140 mg/dL and 200 mg/dL [29].

Metformin (Loditon, Standard Chem and Pharm, Taiwan) was started at 500 mg/day in the first month, 1000 mg/day in the second month, 1500 mg/day in the third month, and continued at 1500 mg/day for one year, as described previously [17]. A decrease to 1000 mg daily dose was allowed for patients who reported gastrointestinal symptoms, such as abdominal distention and cramping, that affected daily activities. Any lowering of doses was recorded as having received a non-maximal regimen. Patients returned for prescriptions every month in the first 6 months, and every 2 months in the last 6 months. Compliance was assessed at every prescription for the preceding interval, based on the patients’ report of retaining missed pills from that interval. The number of intervals with missed pills was normalized by the number of intervals with a prescription, and a rate < 30% was considered compliant. Patients were reevaluated for hormonal, metabolic, anthropometric, hepatic, inflammatory, oxidative markers and blood pressures after 3 (M3), 6 (M6), and 12 months (M12) of treatment.

### Assay methods

Plasma glucose, glutamic oxaloacetic transaminase (GOT), glutamic pyruvic transaminase (GPT), and high sensitivity C-reactive protein (hsCRP) were measured using an autoanalyzer (Toshiba TBA-120 FR; Toshiba, Tokyo, Japan). Serum insulin levels were determined using a microparticle enzyme immunoassay in an AxSYM system (Abbott Laboratories, Dainabot Co., Tokyo, Japan). Serum total testosterone was measured using radioimmunoassays (RIA; Diagnostic Systems Laboratories, Webster, TX, USA), while serum sex hormone–binding globulin (SHBG) was measured using electrochemiluminescence (Elecsys 2010; Roche Diagnostics, Mannheim, Germany). The free androgen index (FAI) was calculated as FAI = total testosterone/SHBG × 100%. The homeostatic model assessment-insulin resistance (HOMA-IR) was calculated as HOMA-IR = (glucose (mg/dl) × 0.05551) × insulin (IU/ml) / 22.5 [30].

Plasma 8-OHdG was assessed using the HT 8-oxo-dG ELISA Kit II (R&D Systems, Inc., Minneapolis, MN, USA; catalog no. 4380–096-K). Peripheral blood leukocyte DNA was extracted from the buffy coat using a QIAamp DNA kit (Qiagen, Valencia, CA, USA). The mtDNA-CN was assessed using quantitative PCR (SYBR green system, Applied Biosystems, Waltham, MA, USA) according to a previous study [31]. The amount of mtDNA-CN was normalized to the expression of nuclear beta-globin. Primers for the mitochondrial ND1 gene were 5′-AACATACCCATGGCCAACCT-3′ and 5′-AGCGAAGGGTTGTAGTAGCCC-3′. Primers for the nuclear beta-globin gene were 5′-GAAGAGCCAAGGACAGGTAC-3′ and 5′-CAACTTCATCCACGTTCACC-3′. The threshold cycle number (Ct) was defined as the number of polymerase chain reaction cycles needed to produce 20 ng of DNA product; mtDNA-CN was calculated using the equation: relative copy number = 2^ΔCt^ (ΔCt = Ct_beta-globin_ − Ct_ND1_).

### Statistical analysis

Baseline comparisons between groups were performed using the Mann–Whitney U test, and the chi-squared test as appropriate. Correlations at baseline were performed using the Spearman’s rank correlation test. The significance of changes in clinical variables at M3, M6, and M12 were compared to the baseline using the paired samples Wilcoxon signed-rank test. The changes in clinical variables with respect to time (in months) under treatment were assessed using the generalized estimating equations (GEE), which are hierarchical regression models that account for repeated measurements [32]. Regressions between the change in mtDNA-CN and changes in other clinical variables were performed using the bivariate GEE model, after normalizing each variable by interval. The normalization step transforms each variable into the logarithm of the magnitude of change in each interval, before modeling with GEE. As GEE is able to model longitudinal data with intermittent missing data, all available data were used for analysis. A two-sided significance level of < 0.05 was considered significant. The data analysis of this study was done in SPSS (SPSS Inc. Released 2008. SPSS Statistics for Windows, Version 17.0. Chicago: SPSS Inc.).

## Results

Baseline characteristics of the patients are summarized in Table 1. The median age at enrollment was 24 years old with a median BMI of 23.2 kg/m^2^, and a median waist circumference of 84 cm. Fifty-one percent of the patients were considered overweight, and 64.8% were abdominally obese. The median fasting glucose was 82.0 mg/dL, and the median HOMA-IR was 0.8. Metabolically, none of the patients had overt diabetes on the 2-h 75 g glucose tolerance test, and 14.8% were considered glucose impaired. The patients had a median FAI of 2%, and a LH/FSH ratio of 1.7. Medication parameters show that 46.4% of patients received a non-maximal regimen, and 61.9% of patients were considered compliant.

**Table 1:** Characteristics of the patients in the cohort

Baseline Parameters	Total (***N*** = 88)	Normal Weight (***N*** = 43)	Overweight (***N*** = 45)	***p***-value between weights
Age (years)	24.0 (20.0–28.0)	24.0 (21.0–26.8)	25.5 (17.3–29.8)	0.697
BMI (kg/m^2^)	23.2 (20.6–29.8)	20.9 (19.2–21.9)	30.6 (25.4–37.4)	< 0.001*
Overweight	51.1% (45/88)	0% (0/43)	100% (45/45)	–
Waist (cm)	84.0 (77.0–94.0)	76.3 (73.6–81.5)	93.8 (86.6–109.0)	< 0.001*
Abdominally obese	64.8% (57/88)	32.6% (14/43)	95.6% (43/45)	< 0.001*
Fasting glucose (mg/dL)	82.0 (78.0–86.8)	80.5 (76.0–84.8)	87.0 (81.3–91.0)	< 0.001*
Fasting insulin (mIU/L)	4.3 (2.0–12.9)	2.0 (2.0–4.7)	13.8 (6.5–21.4)	< 0.001*
HOMA-IR	0.8 (0.4–2.7)	0.4 (0.4–1.0)	2.9 (1.3–4.9)	< 0.001*
75 g GTT 2 h (mg/dL)	105.0 (85.8–129.0)	91.5 (80.3–104.3)	122.5 (115.3–142.3)	< 0.001*
Glucose impaired	14.8% (8/54)	0.0% (0/28)	30.7% (8/26)	0.001*
Testosterone (ng/ml)	0.6 (0.4–0.8)	0.5 (0.3–0.7)	0.6 (0.4–0.9)	0.676
SHBG (nmol/l)	31.1 (19.0–45.0)	42.2 (34.4–59.4)	18.2 (15.2–25.3)	< 0.001*
FAI (%)	2.0 (1.2–3.4)	1.1 (0.8–1.9)	3.5 (2.2–4.4)	< 0.001*
FSH (mIU/ml)	6.1 (5.3–7.4)	6.3 (5.5–7.8)	5.6 (5.1–6.6)	0.037*
LH (mIU/ml)	10.8 (7.7–14.9)	13.8 (10.3–16.7)	9.1 (6.4–10.8)	< 0.001*
LH/FSH ratio	1.7 (1.2–2.5)	2.1 (1.8–2.7)	1.5 (1.2–1.8)	0.001*
GOT (U/L)	19.0 (17.0–23.0)	18.5 (16.0–21.8)	20.5 (19.0–31.3)	0.464
GPT (U/L)	17.0 (12.0–24.0)	13.0 (11.0–17.8)	24.0 (16.0–38.3)	< 0.001*
hsCRP (mg/dL)	0.09 (0.03–0.27)	0.04 (0.02–0.08)	0.31 (0.09–0.63)	< 0.001*
8-OHdG	34.6 (27.7–45.4)	38.5 (30.6–53.9)	31.0 (25.3–42.6)	0.210
mtDNA-CN	55.4 (26.1–100.3)	47.2 (24.8–77.5)	50.7 (23.9–98.6)	0.786
**Medication Parameters**				
Non-maximal regimen	46.4% (39/84)	64.3% (27/42)	28.6% (12/42)	0.001*
Compliant	61.9% (52/84)	61.9% (26/42)	61.9% (26/42)	0.999

Data given as median (Q1-Q3); p-value < 0.05 is denoted with * for comparisons between normal-weight and overweight groups; *BMI* Body mass index, *HOMA-IR* Homeostatic model assessment-insulin resistance, *GTT* Glucose tolerance test, *SHBG* Sex hormone binding globulin, *FAI* Free androgen index, *FSH* Follicle stimulating hormone, *LH* Luteinizing hormone, *GOT/GPT* Glutamic oxaloacetic/pyruvic transaminase, *hsCRP* High sensitivity C-reactive protein, *8-OHdG* 8-hydroxy-2-deoxyguanosine, *mtDNA-CN* Mitochondrial DNA copy number

A stratified analysis based on a BMI cutoff of ≥23 kg/m^2^ shows that a subgroup with relative insulin resistance could be effectively identified. The overweight group was associated with greater waist size, higher fasting glucose, higher fasting insulin, and higher blood glucose on the 2-h 75 g glucose tolerance test. The overweight group also had greater percentages of abdominally obese and glucose impairment. Weight appears to affect whether or not patients received a non-maximal regimen, with 64.3% of normal weight patients and 28.6% of overweight patients having received a lower dose than originally specified (*p* = 0.003). Weight did not affect whether patients were compliant with the prescribed regimen.

At study baseline, mtDNA-CN did not correlate with any of the anthropometric measures, inflammatory markers, metabolic markers, nor with 8-OHdG levels (Table 2). There was no correlation between 8-OHdG and age, BMI, waist circumference, testosterone, SHBG, FAI, fasting insulin, HOMA-IR, 2-h 75 g glucose tolerance test, GOT, SBP, DBP, hsCRP, and mtDNA-CN. There was a negative correlation between fasting glucose and 8-OHdG (r = − 0.25524, *p* = 0.017), and between GPT and 8-OHdG (r = − 0.23377, *p* = 0.029).

**Table 2:** Correlations between mtDNA-CN, 8-OHdG, and clinical variables at baseline

	mtDNA-CN	8-OHdG
Age	− 0.06283 (0.565)	− 0.07736 (0.476)
BMI	− 0.03618 (0.741)	− 0.10975 (0.312)
Waist circumference	− 0.02719 (0.804)	− 0.04646 (0.669)
Testosterone	0.18848 (0.082)	0.10680 (0.325)
SHBG	0.02572 (0.816)	0.17169 (0.116)
FAI	0.12249 (0.267)	−0.11352 (0.301)
Fasting glucose	0.07002 (0.522)	−0.25524 (0.017)*
Fasting insulin	0.02663 (0.808)	−0.02919 (0.788)
HOMA-IR	0.00987 (0.928)	−0.07668 (0.480)
75 g GTT 2 h	0.14033 (0.312)	−0.06693 (0.634)
GOT	−0.01371 (0.900)	−0.15375 (0.155)
GPT	−0.08597 (0.431)	−0.23377 (0.029)*
SBP	−0.02479 (0.821)	0.01901 (0.861)
DBP	−0.09383 (0.390)	−0.00817 (0.940)
hsCRP	−0.12933 (0.290)	−0.06189 (0.608)
8-OHdG	0.06776 (0.538)	–
mtDNA-CN	–	0.06776 (0.538)

Data given as r (p-value); p-value < 0.05 is denoted with *; *BMI* Body mass index, *SHBG* Sex hormone binding globulin, *FAI* Free androgen index, *HOMA-IR* Homeostatic model assessment-insulin resistance, *GTT* Glucose tolerance test, *GOT/GPT* Glutamic oxaloacetic/pyruvic transaminase, *SBP* Systolic blood pressure, *DBP* Diastolic blood pressure, *hsCRP* High sensitivity C-reactive protein, *8-OHdG* 8-hydroxy-2-deoxyguanosine, *mtDNA-CN* Mitochondrial DNA copy number

Over the course of the study, 88 women completed workup at baseline, 85 at 3 months, 87 at 6 months, and 61 at 12 months. Treatment with metformin was associated with significant time-dependent decreases in testosterone, SHBG, FAI, GOT, GPT, SBP, DBP, mtDNA-CN, and 8-OHdG (Table 3). When compared pair-wise with the baseline, BMI, testosterone, FAI and 8-OHdG showed decreases at M3, M6, and M12. Significant decreases were seen in GOT, GPT, DBP, and mtDNA-CN at M6 and M12. SHBG, SBP, and HSCRP were significantly decreased at M12, while fasting glucose and HOMA-IR were decreased at M6 and M3, respectively. No significant change was seen in fasting insulin.

**Table 3:** The change in median values for each variable during the one-year treatment with metformin

	Baseline (N = 88)	3 months (*N* = 85)	6 months (*N* = 87)	12 months (*N* = 61)	Time Estimate (months)	p-value
BMI (kg/m^2^)	23.2 (20.6–29.8)	22.7* (20.7–29.3)	22.7* (20.7–29.0)	22.6* (20.5–29.0)	− 0.048	0.123
Testosterone (ng/ml)	0.61 (0.41–0.81)	0.48* (0.33–0.68)	0.40* (0.26–0.60)	0.36* (0.28–0.52)	− 0.019	< 0.001**
SHBG (nmol/l)	31.1 (19.0–45.0)	30.5 (20.0–46.6)	32.4 (18.8–46.0)	17.3* (17.7–36.7)	− 0.323	0.014**
FAI (%)	6.84 (4.07–11.86)	5.05* (3.53–8.34)	4.27* (2.52–8.34)	5.06* (3.10–7.53)	− 0.178	< 0.001**
Fasting glucose (mg/dL)	82 (78–87)	82 (77–85)	81* (78–85)	84 (79–87)	0.046	0.437
Fasting insulin (mIU/L)	4.27 (2.00–12.93)	3.70 (2.00–11.25)	4.97 (2.00–9.79)	4.28 (2.00–9.04)	− 0.137	0.083
HOMA-IR	0.84 (0.41–2.68)	0.76* (0.39–2.28)	0.95 (0.40–2.07)	0.75 (0.41–1.84)	−0.029	0.103
GOT(U/L)	19 (17–23)	18 (15–23)	17* (15–21)	16* (13–20)	− 0.498	0.002**
GPT (U/L)	17 (12–24)	17 (12–29)	15* (10–23)	12* (9–18)	−0.739	0.012**
SBP (mmHg)	112 (105–124)	112 (104–122)	112 (105–122)	109* (101–118)	−0.251	0.010**
DBP (mmHg)	72 (66–78)	72 (65–79)	71* (65–79)	70* (63–77)	−0.274	< 0.001**
hsCRP (mg/dL)	0.088 (0.038–0.287)	0.099 (0.015–0.262)	0.093 (0.028–0.283)	0.062* (0.026–0.252)	−0.006	0.267
mtDNA-CN	55.38 (26.14–100.27)	34.78 (19.27–78.08)	33.42* (20.45–55.78)	24.11* (16.92–66.75)	−4.509	0.003**
8-OHdG	34.62 (27.70–45.44)	31.95* (23.07–42.12)	29.96* (21.21–39.09)	18.57* (12.13–31.15)	−1.299	< 0.001**

Data given as median (Q1-Q3); * denotes p-value < 0.05 compared with the baseline; ** denotes *p*-value < 0.05 for the time variable; *BMI* Body mass index, *SHBG* Sex hormone binding globulin, *FAI* Free androgen index, *HOMA-IR* Homeostatic model assessment-insulin resistance, *GOT/GPT* Glutamic oxaloacetic/pyruvic transaminase, *SBP* Systolic blood pressure, *DBP* Diastolic blood pressure, *hsCRP* High sensitivity C-reactive protein, *mtDNA-CN* Mitochondrial DNA copy number, *8-OHdG* 8-hydroxy-2-deoxyguanosine

**Fig. 1:**
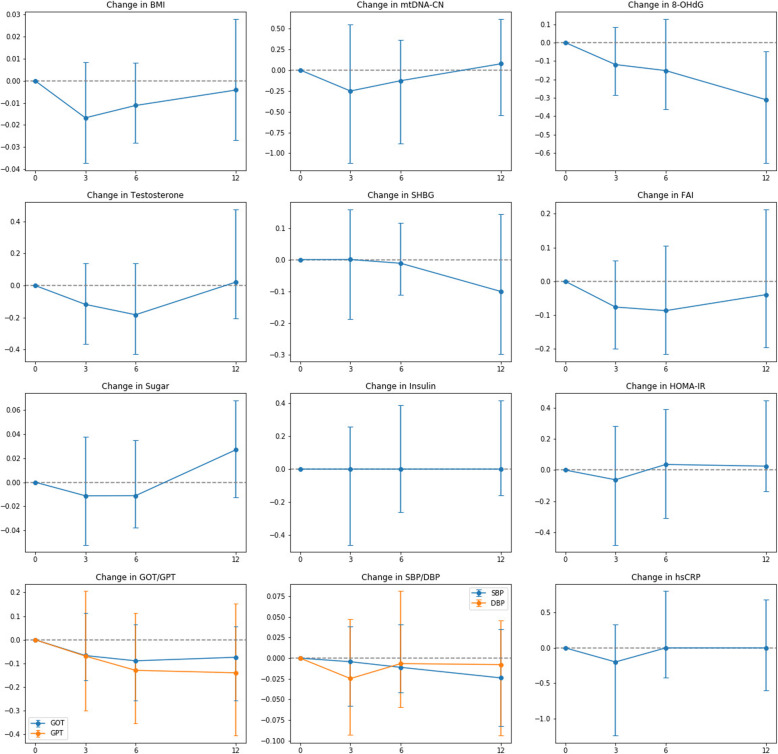
The normalized median values for the change in clinical variables during treatment. The values representing change were normalized by taking the logarithm of the magnitude of change in each interval. Transformed values are zero for no change, positive values for increases, and negative values for decreases. The points on the line represent the medians, and Q1 and Q3 are denoted by the error bars. Abbreviations are as follows: BMI: Body mass index; SHBG: sex hormone binding globulin; FAI: Free androgen index; HOMA-IR: homeostatic model assessment-insulin resistance; GOT/GPT: glutamic oxaloacetic/pyruvic transaminase; hsCRP: high sensitivity C-reactive protein; SBP: systolic blood pressure; DBP: diastolic blood pressure; mtDNA-CN: mitochondrial DNA copy number; 8-OHdG: 8-hydroxy-2-deoxyguanosine

A number of clinical variables were evaluated as possible explanatory factors for the change in mtDNA-CN. Regressions of the change in mtDNA-CN due to changes in other clinical variables were performed. Between-variables comparisons were conducted on normalized values calculated as the logarithm of the magnitude of change (Fig. 1). These transformed values are negative for decreases and positive for increases, and are summarized in the supplement (See Supplementary Table 1, Additional file 1). The strength of the relationships were represented by the coefficient estimates of the explanatory variables in the GEE models (Table 4). From the GEE models, the change in mtDNA-CN appeared unrelated to the changes in BMI, SHBG, FAI, fasting glucose, fasting insulin, HOMA-IR, GOT, GPT, SBP, DBP, hsCRP, or 8-OHdG. There was a sole statistical relationship between the change in mtDNA-CN and the change in testosterone (β = 0.3236, *p* = 0.0357).

**Table 4:** Coefficient estimates for each clinical variable in predicting change in mtDNA-CN

Longitudinal Variables	Estimate	SE	95% CI		p-value
BMI	−2.4737	1.8465	−6.0927	1.1453	0.1803
Testosterone	0.3236	0.1541	0.0216	4.4097	0.0357*
SHBG	0.3269	0.2903	−0.2420	0.8959	0.2600
FAI	0.3530	0.3511	−0.3352	1.0412	0.3147
Fasting glucose	1.5686	1.5010	−1.3734	4.5106	0.2960
Fasting insulin	0.0296	0.1470	−0.2584	0.3177	0.8403
HOMA-IR	0.0427	0.1423	−0.2362	0.3217	0.7641
GOT	0.0387	0.2007	−0.3548	0.4322	0.8471
GPT	−0.1001	0.1347	−0.3642	0.1640	0.4575
SBP	−0.9090	1.1083	−3.0813	1.2633	0.4121
DBP	0.0612	0.7854	−1.4780	1.6005	0.9379
hsCRP	−0.0175	0.0466	−0.1089	0.0738	0.7070
8-OHdG	−0.1829	0.1041	−0.3869	0.0211	0.0789
**Baseline Variables**					
Overweight	0.0583	0.1227	−0.1822	0.2988	0.6345
Abdominal obesity	0.0384	0.1392	−0.2343	0.3111	0.7826
Glucose impairment	0.2663	0.1842	−0.0948	0.6275	0.1483
**Medication Variables**					
Non-maximal dose	−0.0421	0.1241	−0.2853	0.2012	0.7346
Compliant	0.1022	0.1300	−0.1526	0.3570	0.4318

p-value < 0.05 is denoted with*; *BMI* Body mass index, *SHBG* Sex hormone binding globulin, *FAI* Free androgen index, *HOMA-IR* Homeostatic model assessment-insulin resistance, *GOT/GPT* Glutamic oxaloacetic/pyruvic transaminase, *SBP* Systolic blood pressure, *DBP* Diastolic blood pressure, *hsCRP* High sensitivity C-reactive protein, *8-OHdG* 8-hydroxy-2-deoxyguanosine

The change in mtDNA-CN due to other patient factors, such as overweight, abdominally obese, glucose impaired, non-maximal regimen, and compliant status was examined. The change in mtDNA-CN was unrelated to any of these factors (Table 4). We also tested whether compliance affected the changes in other clinical variables (See Supplementary Table 2, Additional file 1). There were no relations between compliance and the change in any of the clinicial variables.

From the longitudinal plots of the variables (Fig. 1), a few variable pairs appeared to have similar changes in median values over time, including GOT/GPT, SBP/DBP, HOMA-IR/hsCRP, and 8-OHdG/SHBG pairs. We assessed for associations between these pairs using the GEE models. The relationships between GOT and GPT (β = 0.5131, *p* < 0.0001), and between SBP and DBP (β = 0.0418, *p* < 0.0001) were significant, while the relationships between HOMA-IR and hsCRP, and between 8-OHdG and SHBG were not. This perceived discrepancy between the GEE results and the visual interpretation of the longitudinal plots can be explained by the contribution of subject-level interactions, which are tested by the GEE models. In essence, although the changes in HOMA-IR/hsCRP and 8-OHdG/SHBG pairs may seem similar, judging by sample medians, this may not reflect real changes in the individual. The changes in HOMA-IR/hsCRP and 8-OHdG/SHBG pairs were not statistically significant by this assessment.

## Discussion

As an insulin sensitizer, metformin was originally used to treat insulin resistance associated with PCOS [15]. Since then, the drug has been extensively studied, and has shown activity against many clinical derangements in patients with PCOS, ranging from obesity, hypertension to metabolic, endocrine and menstrual dysfunctions [16, 17]. Metformin has also shown activity in reducing oxidative end-products, including malondialdehyde and 8-OHdG [22, 23]. As the production of oxidative end-products is intimately tied to mitochondrial functions [6], the effects of metformin on mitochondria functions were explored in one study [8]. In the study, metformin improved mitochondrial respiration capacity, energy utilization, and mitochondria mass in women with PCOS [8]. However, the study provided no data on the change in mtDNA-CN, which has been frequently utilized as a marker of mitochondria dysfunction [33, 34].

This study evaluates the longitudinal change in mtDNA-CN in women with PCOS, who were treated with metformin. In this study, a progressive, time-dependent decrease in mtDNA-CN was observed in patients treated with metformin. However, the nature of this decrease in mtDNA-CN is not immediately clear, as the literature diverges on the relationship between mtDNA-CN and diseases. In cross-sectional studies, mtDNA-CN was lower in diseases such as multiple sclerosis and Parkinson’s disease [13, 14], while others have found lower levels of mtDNA-CN in normal pregnancies, when compared with pregnancies complicated with gestational diabetes [35]. Although no conclusion can be drawn on the role of mtDNA-CN from these studies, mtDNA-CN tends to correspond with changes in oxidative markers, such as 8-OHdG [35–37]. As 8-OHdG is produced during ROS-mediated DNA damages, it is often utilized as a marker of intra-cellular oxidative stress [24]. Due to its associations with 8-OHdG, many studies have similarly ascribed a decrease in mtDNA-CN to a decrease in cellular stress and damages [10, 11, 38].

In the absence of an authoritative study on the nature of these mtDNA-CN changes, the difficulty lies in reconciling the decrease in mtDNA-CN associated with PCOS [9], and the decrease in mtDNA-CN associated with metformin treatment in these same patients. In both situations, however, 8-OHdG appears to corroborate with the change in mtDNA-CN. Both 8-OHdG and mtDNA-CN are lower in patients with PCOS [9, 39], and lower with metformin treatment [23]. As no causal research is currently available, any explanation is speculative, but these correlations do point to an improved oxidative state. The means through which 8-OHdG and mtDNA-CN are reduced in PCOS is unknown. In the absence of studies on the nature of these mtDNA-CN changes, it may be prudent for clinicians to remain wary of possible adverse effects from metformin on energy metabolism in these patients.

In this study, we also examined if a statistical relationship can be established between mtDNA-CN and 8-OHdG. When assessed with regression, the change in 8-OHdG did not contribute significantly to the change in mtDNA-CN. This shows that although the markers are linked clinically, they are not correlated when assessed in a measurement-by-measurement basis. In fact, mtDNA-CN and 8-OHdG differed in their associations with patient characteristics. At study baseline, mtDNA-CN was uncorrelated with metabolic factors, while 8-OHdG was correlated with fasting glucose and GPT. The significant association with GPT and not with GOT may be explained by the greater sensitivity of GPT for metabolic syndrome [40], which also affects 8-OHdG [41].

The change in mtDNA-CN was also uncorrelated with most other clinical variables, including BMI, which was implicated in previous studies [9, 42]. Only testosterone appears to be statistically related to the change in mtDNA-CN, signifying that the changes in mtDNA-CN and testosterone were correspondent during treatment with metformin. A literature search for this possible relationship found no human studies, but two murine studies were found where mtDNA-CN levels in skeletal muscles correlated with testosterone exposures [43, 44]. We found no impact from receiving a non-maximal regimen, but this could be due to the greater number of patients receiving a non-maximal regimen in normal weight patients. Compliance measure in this study also did not affect changes in mtDNA-CN or any of the other clinical factors. This is probably because patients returned to the clinic for prescriptions every 1 to 2 months, which ensured that there were continued use of medications, and very few pills were actually missed.

There are a few limitations to this study. Although we are currently the only group to demonstrate a change in mtDNA-CN during treatment with metformin in patients with PCOS, this study is a longitudinal cohort by design. A placebo-treated, age-matched control that matches the study group is needed to prove cause and effect, but we feel that this cannot be justified at this time due to a lack of previously reported interaction between metformin and mtDNA-CN. In addition, there is an ethical concern from withholding metformin when it has been proven effective in randomized control trials [45]. Secondly, although the cohort was identified using the Rotterdam criteria for PCOS [25], and we were able to identified a subset of patients with insulin resistance to explore its relationship to mtDNA-CN, our group is younger and had a glucose impaired rate of 14.8%, compared with the 64.4% in a previous report [46]. This may affect the applicability of the study results to groups with more insulin resistance. Lastly, although 98.9% (87/88) of patients returned at 6-month, there will inevitably be concerns about the lower number of patient remaining at 1-year (61/88). It should be noted that the data at 1-year was not used alone in statistical tests, but was part of hierarchical regression models (GEE). As the hierarchical regression model has been shown to be resistant to intermittently missing data [47], the results should remain valid as long as the data was missing at random. We checked this assumption by assessing for bias between patient characteristics, included anthropometric, hormonal, metabolic measures, factors such as non-maximal regimen, medical compliance, and whether these affected patient remainingin the study at 1-year (See Supplementary Table 3, Additional file 1). None of the tested variables were related to whether patients remained in the study.

## Conclusion

Time-dependent decreases in mtDNA-CN, 8-OHdG, testosterone, SHBG, and hepatic enzymes were seen in a longitudinal observational study in patients with PCOS, who received metformin for 1 year. The decrease in mtDNA-CN likely reflects an improvement in cellular oxidative stress, judging by the concurrent decrease in 8-OHdG. However, there is currently no causal research on the nature of these mtDNA-CN reductions. Clinicians should, therefore, remain cautious about the use of metformin as it does appear to alter mitochondria functions in these patients. We found no association between overweight, abdominal obesity, insulin resistance, patient compliance and the change in mtDNA-CN. Testosterone was correlated with the change in mtDNA-CN, and may have a role in modulating the effect of metformin on the mitochondria, but more work is needed to ascertain this relationship.

## Supplementary information

**Additional file 1 Supplementary Tables*.*Supplementary Table 1.** The normalized changes for each variable over the duration of the study. **Supplementary Table 2*.*** Coefficient estimates for medical compliance in explaining change in each clinical variable. **Supplementary Table 3*.*** Coefficient estimates for each clinical variable in predicting patient drop-out from the *logistic regression* models.

## Data Availability

The datasets used during the current study are available from the corresponding author on reasonable request.

## Abbreviations

PCOS: Polycystic ovarian syndrome
mtDNA-CN: Mitochondria DNA copy number
8-OHdG: 8-hydroxydeoxyguanosine
ROS: Reactive oxygen species
BMI: Body mass index
SBP: Systolic blood pressure
DBP: Diastolic blood pressure
GOT: Glutamic oxaloacetic transaminase
GPT: Glutamic pyruvic transaminase
hsCRP: High sensitivity C-reactive protein
FAI: Free androgen index
HOMA-IR: Homeostatic model assessment-insulin resistance
SHBG: Serum sex hormone–binding globulin
GEE: Generalized estimating equations
