# Supplementary material for: Changes in peripheral mitochondrial DNA copy number in metformin-treated women with polycystic ovary syndrome: a longitudinal study
Source: Reprod Biol Endocrinol. 2020 Jul 13;18:69. doi: 10.1186/s12958-020-00629-5 (PMC7359290; doi:10.1186/s12958-020-00629-5)
Supplement: Supplementary file 1 — Additional file 1 Supplementary Tables.Supplementary Table 1. The normalized changes for each variable over the duration of the study. Supplementary Table 2. Coefficient estimates for medical compliance in explaining change in each clinical variable. Supplementary Table 3. Coefficient estimates for each clinical variable in predicting patient drop-out from the logistic regression models. [file 12958_2020_629_MOESM1_ESM.docx]

**Supplementary Table 1.** The normalized changes for each variable over the duration of the study.

|  | Baseline  (N = 88) | 3 months  (N = 85) | 6 months  (N = 87) | 12 months  (N = 61) |
| --- | --- | --- | --- | --- |
| BMI | 0 | -0.0168 (-0.0374-0.0084) | -0.0112 (-0.0281-0.008) | -0.0042 (-0.0268-0.0281) |
| Testosterone | 0 | -0.1177 (-0.3624-0.1383) | -0.1823 (-0.4274-0.1376) | 0.0215 (-0.2047-0.4735) |
| SHBG | 0 | 0.0002 (-0.1879-0.1582) | -0.0116 (-0.1114-0.1153) | -0.1009 (-0.2987-0.1438) |
| FAI | 0 | -0.0763 (-0.1999-0.0618) | -0.0869 (-0.2155-0.1043) | -0.0395 (-0.1954-0.2125) |
| Fasting glucose | 0 | -0.0113 (-0.0521-0.0377) | -0.0112 (-0.0376-0.0352) | 0.0270 (-0.0124-0.0681) |
| Fasting insulin | 0 | 0.0000 (-0.4606-0.2568) | 0.0000 (-0.2622-0.3868) | 0.0000 (-0.1620-0.4160) |
| HOMA-IR | 0 | -0.0625 (-0.4810-0.2813) | 0.0359 (-0.3082-0.3901) | 0.0253 (-0.1381-0.4485) |
| GOT | 0 | -0.0668 (-0.1719-0.1122) | -0.0890 (-0.2556-0.0645) | -0.0741 (-0.2578-0.0572) |
| GPT | 0 | -0.0690 (-0.2986-0.2058) | -0.1293 (-0.3523-0.1129) | -0.1398 (-0.4055-0.1542) |
| SBP | 0 | -0.0043 (-0.0576-0.0381) | -0.0111 (-0.0413-0.0404) | -0.0239 (-0.0820-0.0350) |
| DBP | 0 | -0.0247 (-0.0928-0.0470) | -0.0066 (-0.0596-0.0812) | -0.0078 (-0.0938-0.0453) |
| hsCRP | 0 | -0.1991 (-1.2426-0.3322) | 0.0000 (-0.4247-0.8045) | 0.0000 (-0.6038-0.6882) |
| mtDNA-CN | 0 | -0.2520 (-1.1209-0.5449) | -0.1282 (-0.8844-0.3590) | 0.0754 (-0.5460-0.6164) |
| 8-OHdG | 0 | -0.1199 (-0.2864-0.0819) | -0.1526 (-0.3627-0.1293) | -0.3113 (-0.6570--0.0495) |

Data given as median (Q1-Q3); BMI: Body mass index; SHBG: sex hormone binding globulin; FAI: Free androgen index; HOMA-IR: homeostatic model assessment-insulin resistance; GOT/GPT: glutamic oxaloacetic/pyruvic transaminase; SBP: systolic blood pressure; DBP: diastolic blood pressure; hsCRP: high sensitivity C-reactive protein; mtDNA-CN: mitochondrial DNA copy number; 8-OHdG: 8-hydroxy-2-deoxyguanosine.

**Supplementary Table 2.** Coefficient estimate for medical compliance in explaining change in each clinical variable.

|  | Estimate | | SE | | 95% CI | | p-value | |
| --- | --- | --- | --- | --- | --- | --- | --- | --- |
| BMI | -0.0049 | 0.0045 | | -0.0137 | | 0.0039 | | 0.2745 |
| Testosterone | 0.0009 | 0.0423 | | -0.0820 | | 0.0837 | | 0.9835 |
| SHBG | 0.0259 | 0.0234 | | -0.0200 | | 0.0718 | | 0.2684 |
| FAI | -0.0171 | 0.0516 | | -0.1182 | | 0.0840 | | 0.7408 |
| Fasting glucose | -0.0043 | 0.0060 | | -0.0162 | | 0.0075 | | 0.4756 |
| Fasting insulin | -0.0147 | 0.0725 | | -0.1568 | | 0.1274 | | 0.8389 |
| HOMA-IR | -0.0198 | 0.0730 | | -0.1629 | | 0.1234 | | 0.7867 |
| GOT | 0.0452 | 0.0400 | | -0.0333 | | 0.1237 | | 0.2590 |
| GPT | 0.0506 | 0.0507 | | -0.0486 | | 0.1499 | | 0.3174 |
| SBP | -0.0018 | 0.0071 | | -0.0156 | | 0.0120 | | 0.7973 |
| DBP | -0.0082 | 0.0104 | | -0.0285 | | 0.0121 | | 0.4278 |
| hsCRP | -0.0631 | 0.1583 | | -0.3734 | | 0.2473 | | 0.6904 |
| 8-OHdG | -0.0377 | 0.0539 | | -0.1433 | | 0.0679 | | 0.4845 |
| mtDNA-CN | 0.1022 | 0.1300 | | -0.1526 | | 0.3570 | | 0.4318 |

p-value < 0.05 is denoted with*; BMI: Body mass index; SHBG: sex hormone binding globulin; FAI: Free androgen index; HOMA-IR: homeostatic model assessment-insulin resistance; GOT/GPT: glutamic oxaloacetic/pyruvic transaminase; SBP: systolic blood pressure; DBP: diastolic blood pressure; hsCRP: high sensitivity C-reactive protein; 8-OHdG: 8-hydroxy-2-deoxyguanosine; mtDNA-CN: mitochondrial DNA copy number.

**Supplementary Table 3.** Coefficient estimate for each clinical variable in predicting patient drop-out from the logistic regression models.

| Factor | Estimate | SE | 95% CI | | p-value |
| --- | --- | --- | --- | --- | --- |
| Age | -0.0371 | 0.0434 | 0.8849 | 1.0492 | 0.9636 |
| BMI | 0.0338 | 0.0391 | 0.9580 | 1.1168 | 0.3877 |
| Waist | -0.0244 | 0.0187 | 0.9407 | 1.0124 | 0.1935 |
| Overweight | 0.1725 | 0.4627 | 0.4798 | 2.9429 | 0.7092 |
| Abdominal obese | 0.5701 | 0.4759 | 0.6959 | 4.4940 | 0.2309 |
| Fasting glucose | -0.0092 | 0.0326 | 0.9296 | 1.0561 | 0.7778 |
| Fasting insulin | 0.0064 | 0.0223 | 0.9633 | 1.0514 | 0.7748 |
| HOMA-IR | 0.0147 | 0.0147 | 0.8326 | 1.2369 | 0.8845 |
| 75g GTT 2hrs | -0.0012 | 0.0106 | 0.9782 | 1.0197 | 0.9080 |
| Glucose impaired | -0.5306 | 0.8038 | 0.1217 | 2.8427 | 0.5092 |
| Testosterone | 0.2046 | 0.7305 | 0.2932 | 5.1362 | 0.7794 |
| SHBG | 0.0166 | 0.0123 | 0.9924 | 1.0416 | 0.1786 |
| FAI | -0.0202 | 0.0377 | 0.9102 | 1.0550 | 0.5910 |
| GOT | -0.0054 | 0.0129 | 0.9698 | 1.0201 | 0.6740 |
| GPT | -0.0024 | 0.0076 | 0.9828 | 1.0126 | 0.7496 |
| SBP | -0.0052 | 0.0166 | 0.9630 | 1.0277 | 0.7554 |
| DBP | -0.0027 | 0.0213 | 0.9566 | 1.0398 | 0.9000 |
| hsCRP | 0.9068 | 0.8878 | 0.4347 | 14.1095 | 0.3070 |
| mtDNA-CN | 0.1635 | 0.5346 | 0.4130 | 3.3580 | 0.7597 |
| 8-OHdG | -0.0067 | 0.0137 | 0.9671 | 1.0204 | 0.6266 |
| Non-maximal regimen | -0.4877 | 0.4807 | 0.2393 | 1.5753 | 0.3103 |
| Compliant | 0.3874 | 0.4648 | 0.5924 | 3.6634 | 0.4045 |

p-value < 0.05 is denoted with*; BMI: Body mass index; HOMA-IR: homeostatic model assessment-insulin resistance; GTT: glucose tolerance test; SHBG: sex hormone binding globulin; FAI: Free androgen index; GOT/GPT: glutamic oxaloacetic/pyruvic transaminase; SBP: systolic blood pressure; DBP: diastolic blood pressure; hsCRP: high sensitivity C-reactive protein; mtDNA-CN: mitochondrial DNA copy number; 8-OHdG: 8-hydroxy-2-deoxyguanosine.
